# Elevated hemoglobin levels in renal transplant recipients with polycystic kidney disease versus other etiologies: exploring mechanisms and implications for outcomes

**DOI:** 10.1007/s40620-023-01868-6

**Published:** 2024-03-01

**Authors:** Yael Rothem, Enosh Askenasy, Maya Siman-Tov, Yana Davidov, Tomer Hoffman, Eytan Mor, Tammy Hod

**Affiliations:** 1 Faculty of Medicine, Tel-Aviv University, Tel Aviv, Israel; 2 Renal Transplant Center, Sheba Medical Center, Tel Hashomer, Israel; 3 Department of Emergency and Disaster Management, School of Public Health, Tel-Aviv University, Tel Aviv, Israel; 4 Liver Disease Center, Sheba Medical Center, Tel Hashomer, Israel; 5 Infectious Diseases Unit, Sheba Medical Center, Tel Hashomer, Israel

**Keywords:** Polycystic kidney disease, Renal transplant, Hemoglobin levels, Native nephrectomy, Patient survival, Allograft function

## Abstract

**Background:**

Autosomal dominant polycystic kidney disease (ADPKD)-related end-stage kidney disease (ESKD) often necessitates transplantation. However, the impact of ADPKD on post-transplant outcomes, specifically hemoglobin levels, remains unknown.

**Methods:**

We retrospectively analyzed 513 Kidney Transplant Recipients (KTRs), of whom 81 had ESKD due to ADPKD (20 with pre-transplant native nephrectomy and 61 without). Hemoglobin levels were evaluated at multiple time intervals post-transplant.

**Results:**

Kidney transplant recipients with ADPKD vs. KTRs with ESKD due to other causes exhibited significantly higher hemoglobin levels in repeated measurement analysis. Multivariable analyses confirmed ADPKD as an independent predictor for elevated hemoglobin levels. In a multivariable logistic regression analysis, the odds for maximum hemoglobin > 15 mg/dL at 3–12 months post-transplant were more than twice as high in ADPKD patients vs. all the other KTRs (Odds Ratio [OR] 2.31, 95% Confidence Interval [CI] 1.3–4.13, *p* < 0.001). Pre-transplant native nephrectomy revealed a trend toward lower hemoglobin levels. Elevated hemoglobin levels were linked to improved estimated glomerular filtration rate (eGFR) at one year post-transplant. Patient survival was enhanced among KTRs with ADPKD compared to other ESKD causes.

**Conclusions:**

Kidney transplant recipients with ADPKD exhibited elevated hemoglobin levels post-transplant, possibly due to prolonged native kidney erythropoietin production. These elevated hemoglobin levels were linked to improved outcomes, including allograft function and patient survival. Future research should further investigate the underlying mechanisms driving favorable ADPKD KTR outcomes.

**Graphical abstract:**

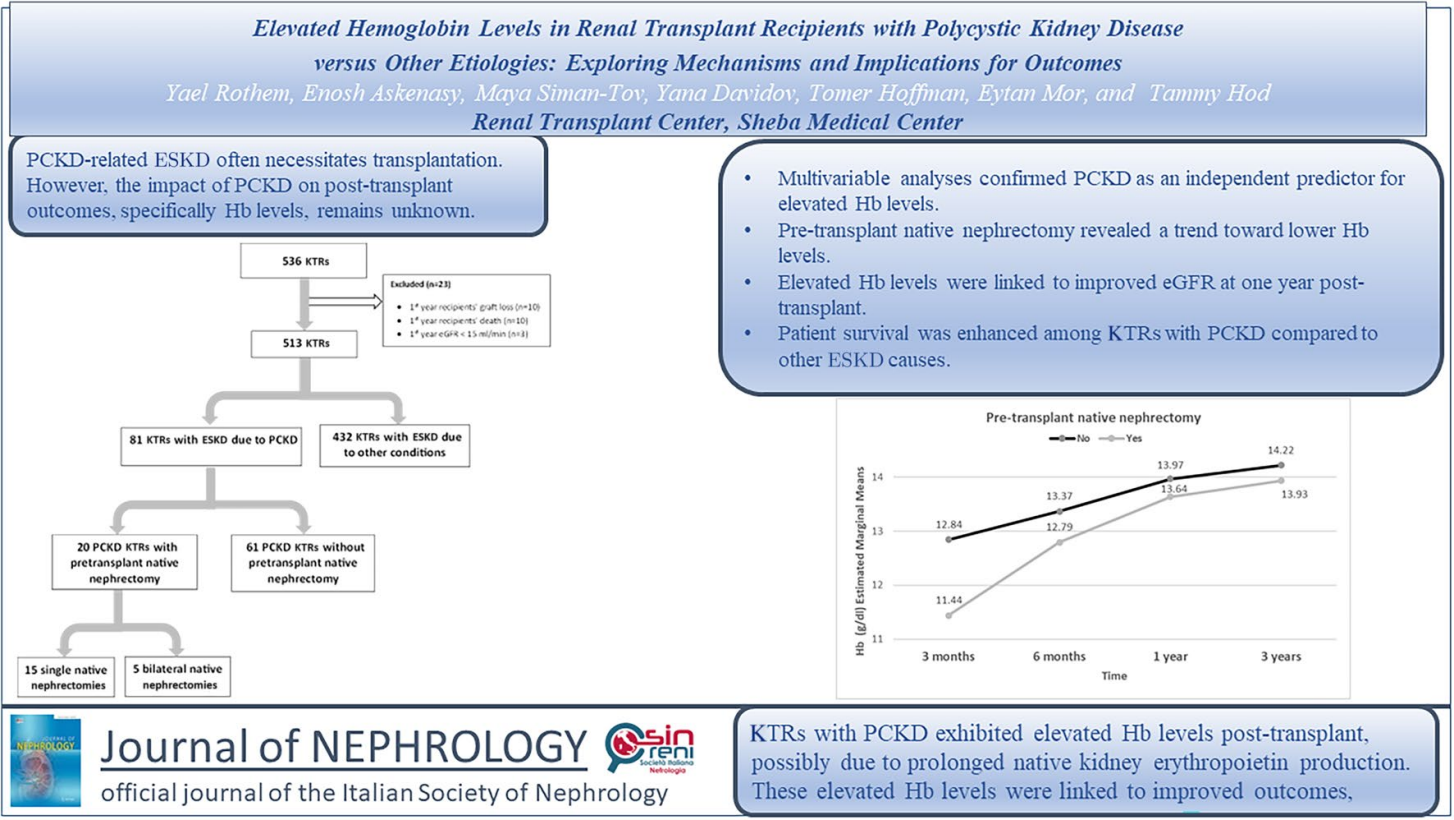

## Introduction

Polycystic kidney disease is an inherited disorder characterized by the progressive formation of fluid-filled cysts that gradually replace normal kidney tissue. It is one of the main causes of end-stage kidney disease (ESKD), often requiring dialysis or kidney transplantation [1]. The primary forms of polycystic kidney disease are recessive and autosomal dominant polycystic kidney disease (ADPKD), with the latter being the prevailing genetic kidney disorder, occurring with a prevalence of 1 in 1000 cases [2].

Patients suffering from ESKD often develop anemia, which is caused by the reduced production of erythropoietin due to kidney damage. Studies have shown that individuals with renal insufficiency secondary to ADPKD tend to have milder anemia than those with renal insufficiency caused by other conditions [3–5]. Furthermore, average serum erythropoietin levels in ADPKD patients are typically approximately double those of patients with ESKD stemming from other etiologies [6]. For example, among 259 ADPKD patients with chronic kidney disease (CKD), hemoglobin levels were notably higher in CKD stages 3 and 4 than in non-PKD CKD patients. However, as CKD progresses, the impact of erythropoietin produced by renal cysts may diminish due to uremia, which suppresses the bone marrow’s response to erythropoietin, resulting in decreased hemoglobin levels [4].

Although ADPKD is one of the most prominent causes of renal transplantation, there is a lack of knowledge regarding post-transplant hemoglobin levels in kidney transplant recipients (KTRs) with ADPKD compared to those with other causes of ESKD. It is known that post-transplantation erythrocytosis is associated with ADPKD, with several mechanisms contributing to the development of the post-transplantation erythrocytosis, including increased erythropoietin production and activation of the renin angiotensin system due to renal structural changes [7, 8]. However, the impact of the native kidneys on erythropoietin production and hemoglobin levels after kidney transplantation remains unclear. Furthermore, previous studies have not rigorously examined the influence of transplant-specific or ADPKD-related factors on post-transplant outcomes in ADPKD patients.

To explore the connection between ADPKD, hemoglobin levels and post-transplant outcomes, this study aimed to compare KTRs with ADPKD to those with different causes of ESKD, including nephrosclerosis, diabetic nephropathy, and glomerulonephritis. Additionally, to elucidate the possible contribution of the native kidneys in KTRs with ADPKD on hemoglobin levels post-transplant, the study examined hemoglobin levels in ADPKD recipients who had undergone pre-transplant native nephrectomy compared to those who had not.

### Methods

### Study population and design

Clinical and biochemical parameters of KTRs were collected retrospectively from the MDClone system, the data acquisition tool at Sheba Medical Center. Additional data were collected from clinical records, as needed. The study was approved by the local ethics committee (IRB approval number: SMC-70–5320).

**Fig. 1:**
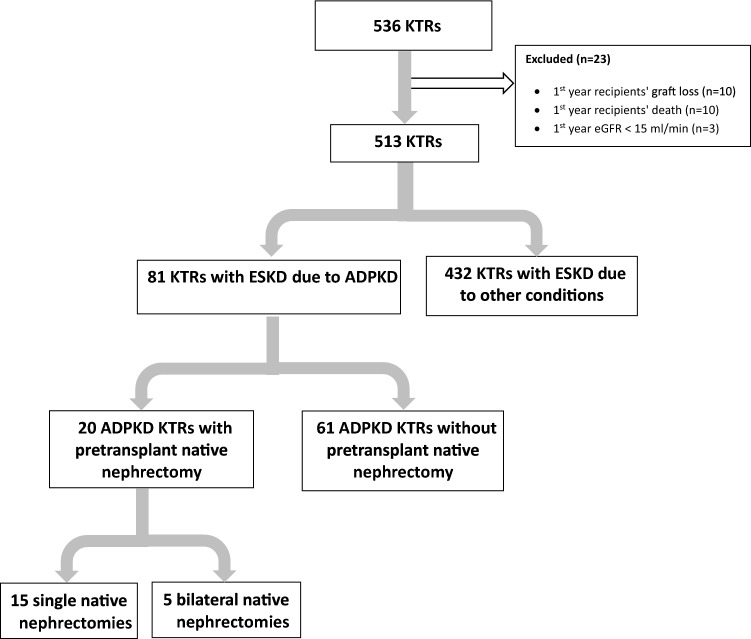
Consort diagram

The initial dataset included 536 KTRs who had undergone kidney transplantation between September 2000 and October 2022. Twenty three KTRs were subsequently excluded from the analysis of the initial dataset, i.e., 10 KTRs with first year graft loss, 10 who died during the first year post-transplant, and 3 due to first year estimated glomerular filtration rate (eGFR) < 15 ml/min. The final study cohort thus comprised 513 KTRs, who are followed up at the renal transplant clinic of Sheba Medical Center. The cohort was divided into patients with ESKD secondary to ADPKD (*n* = 81) and those with ESKD due to other conditions (*n* = 432). Autosomal dominant polycystic kidney disease KTRs were further divided into those who had undergone pre-transplant native nephrectomy (*n* = 20, of whom 15 had undergone single native nephrectomy, and 5 bilateral native nephrectomy) and those who had not undergone such a procedure pre-transplant (*n* = 61) (Fig. 1).

### Immunosuppression

At our medical center, the standard maintenance immunosuppression regimen for KTRs comprises a calcineurin inhibitor (usually tacrolimus), an anti-metabolite (usually a mycophenolate-based drug, mainly mycophenolic acid], and prednisone, as described previously [9]. For KTRs with a low immunological risk of rejection, early steroid withdrawal is implemented 5–8 days after transplant, and the maintenance regimen thus consists of tacrolimus and mycophenolic acid. Conversion to a mammalian target of rapamycin inhibitor (sirolimus or everolimus) is instituted according to the patient’s risk of malignancy and lack of tolerance to calcineurin inhibitors.

### Primary and secondary outcomes

The primary outcome was the mean hemoglobin level 3–12 months post-transplant. To eliminate the effect of changes in immunosuppressive medications or in renal allograft function early post-transplant secondary to ischemic/reperfusion injury, slow and/or delayed graft function, infections and early rejections, we examined hemoglobin levels starting from 3 months post-transplant. Additionally, we examined secondary outcomes, including: eGFR at 1 year post-transplant, allograft loss and patient death, and hemoglobin levels at different time points post-transplant in KTRs with ADPKD versus those with other etiologies of ESKD.

### Data extraction and study assessments

The following information was extracted from electronic patient records: age, sex, etiology of ESKD, dialysis pre-transplant, donor sex and type, transplant date and number, date of allograft loss, and relevant medical history, specifically smoking status, hypertension, congestive heart failure, ischemic heart disease, and pre-transplant diabetes.

The following clinical and biochemical parameters were retrieved in an automated fashion from MDClone: date of death, average systolic and diastolic blood pressures 1–12 months post-transplant, weight and body mass index (BMI), average serum creatinine at 2.5–3.5, 5–7, 10–14, 33–39, 54–66 and 114–126 months post-transplant, average hemoglobin at 0–3, 2.5–3.5, 5–7, and 10–14 months post-transplant, and maximum, minimum and average serum creatinine, hemoglobin and hematocrit 3–12 months post-transplant. The following additional data were retrieved from 3 to 12 months’ post-transplant: average protein/creatinine ratio, maximum, minimum and average tacrolimus trough level, average total white blood cell count, average absolute lymphocyte count, average absolute neutrophil count, average mean corpuscular volume, average mean corpuscular hemoglobin and mean corpuscular hemoglobin concentration, average platelet count, average thyroid stimulating hormone, folic acid, vitamin B12, total bilirubin, lactate dehydrogenase, iron, transferrin and ferritin, and average albumin and globulin. Use of the following medications between 1 and 12 months post-transplant was automatically obtained from MDClone: tacrolimus, cyclosporine, mycophenolic acid, prednisone, mammalian target of rapamycin inhibitors, iron (*per os*), beta blockers, calcium channel blockers and renin angiotensin aldosterone system inhibitors.

### Statistical analysis

All demographic, clinical, and biochemical covariates of interest were systematically tabulated and compared between KTRs with ADPKD and those with ESKD resulting from other causes. Categorical variables were compared using the Chi-squared test, with Fisher’s exact test being used in cases of small cell counts. Continuous variables were subjected to preliminary normality testing using the Shapiro–Wilk test, alongside assessments for equality of variances. Subsequently, normally distributed variables were compared using t-tests or analysis of variance (ANOVA), while non-normally distributed variables were subjected to non-parametric tests.

For the primary outcome of post-transplant hemoglobin levels, both linear and logistic multivariable models were employed. Initially, univariate models were evaluated, with variables displaying significance (*p* < 0.05) or deemed clinically relevant progressing to multivariate modeling. To account for multiple comparisons, a repeated measurements analysis was executed using Bonferroni adjustments, specifically for exploring longitudinal post-transplant trends in hemoglobin and eGFR levels.

Linear regression analysis was also employed to investigate the relationship between eGFR post-transplant and various predictor variables. Survival analyses were conducted to examine patient mortality and allograft loss, utilizing Kaplan–Meier estimation to visualize survival probabilities over time and multivariable Cox regression hazard model for mortality. Statistical analyses were performed using the SPSS software package.

## Results

### Characteristics of the KTR cohort

For the 513 KTRs comprising our study cohort (Table 1), mean transplant age was 54.9 ± 13.4 years and 361 (70.4%) were males. In addition, 332/477 (69.9%) KTRs for whom data were available were on renal replacement therapy before the transplant; and 389/500 (77.8%) had a living donor renal transplant. Past medical histories of hypertension, diabetes, ischemic heart disease and congestive heart failure were recorded for 230 (44.8%), 91 (17.7%), 61 (11.9%) and 29 (5.7%) KTRs, respectively.

**Table 1:** Demographic and clinical characteristics of KTRs stratified by ESKD etiology

Variable	Total cohort (*N* = 513)	ADPKD (*N* = 81)	Other (*N* = 432)	*p*-value
KTR characteristics				
Transplant age (years), [mean ± SD]	54.9 ± 13.4	54.8 ± 11.2	54.9 ± 13.7	0.465
Male sex, *n* (%)	361 (70.4%)	48 (59.3%)	313 (72.5%)	0.017*
Transplant type, *n* = 513 (%)				
Kidney	502 (97.8%)	79 (97.5%)	423 (97.9%)	0.474
Heart kidney	6 (1.2%)	0	6 (1.4%)	
Kidney pancreas	1 (0.2%)	0	1 (0.2%)	
Liver kidney	4 (0.8%)	2 (2.5%)	2 (0.5%)	
Transplant number, *n* = 513 (%)				
1	466 (90.8%)	76 (93.8%)	390 (90.3%)	0.301
2	35 (6.8%)	5 (6.2%)	30 (6.9%)	
3	12 (2.3%)	0	12 (2.8%)	
Smoking status, *n* = 491 (%)				
Current smokers	57 (11.6%)	10 (13.0%)	47 (11.3%)	0.492
Past smokers	94 (19.1%)	11 (14.3%)	83 (20.0%)	
No	341 (69.3%)	56 (72.7%)	285 (68.7%)	
Medical history, *n* = 513 (%)				
HTN	230 (44.8%)	29 (35.8%)	201 (46.5%)	0.075
Pre-transplant diabetes	91 (17.7%)	2 (2.5%)	89 (20.6%)	< .001**
IHD	61 (11.9%)	4 (4.9%)	57 (13.2%)	0.035*
CHF	29 (5.7%)	3 (3.7%)	26 (6.0%)	0.408
Donor type, n = 500 (%)				
Deceased	111 (22.2%)	23 (29.1%)	88 (20.9%)	0.107
Living	389 (77.8%)	56 (70.9%)	333 (79.1%)	
Male	130 (64.7%)	15 (51.7%)	115 (66.9%)	0.115
Female	71 (35.3%)	14 (48.3%)	57 (33.1%)	
Pre-transplant dialysis, n = 477 (%)	332 (69.6%)	51 (67.1%)	281 (70.1%)	0.606

*CHF* congestive heart failure; *HTN* hypertension; *IHD* ischemic heart disease; *KTR* kidney transplant recipients; *SD* standard deviation

^*^ *p* < 0.05; ^**^ p < 0.01

### Univariate comparison of KTRs with ESKD secondary to ADPKD vs. those with ESKD due to other conditions

The KTR cohort was divided into two groups, 81 (15.8%) with ESKD secondary to ADPKD and 432 (84.2%) with ESKD due to other etiologies. There were more males in the group with ESKD due to other conditions compared to KTRs with ADPKD (72.5% vs. 59.3%), and the former group had higher rates of pre-transplant diabetes (20.6% vs. 2.5%) and ischemic heart disease (13.2% vs. 4.9%). All other comparisons of characteristics, including age, transplant type and number, smoking status, donor type and pre-transplant dialysis, are shown in Table 1.

**Fig. 2:**
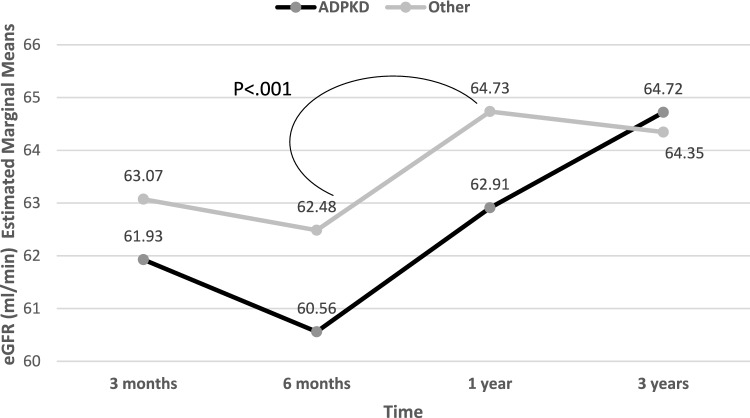
Repeated measurement analysis for eGFR post-transplant in KTRs with ADPKD versus those with other ESKD etiologies. Time effect F _(df=3,762)_ = 3.62 p = .013. Based on pairwise comparisons: the significant was found only among 'Other' group p < .001. Interaction effect F _(df=3,762)_ = 0.56, *p* 0.639

Mean systolic blood pressure was lower and mean diastolic blood pressure was higher in KTRs with vs. without ADPKD (129.5 ± 12.6 vs. 132.8 ± 14.6 mmHg, *p* = 0.05 and 79.1 ± 8.5 vs. 76.3 ± 7.7 mmHg, respectively,* p* = 0.005). Body mass index was also lower in KTRs with ADPKD compared to those without (25.9 ± 3.7 vs. 27.3 ± 4.8 kg/m^2^,* p* = 0.012). There were no significant differences in Chronic Kidney Disease Epidemiology Collaboration (CKD-EPI) eGFR at different time points post-transplant between the two groups, although there was a tendency for a lower serum creatinine and a higher eGFR in KTRs with vs. without ADPKD. In a repeated measurement analysis for mean eGFR at different time points post-transplant adjusted for multiple comparisons, there was no significant difference in eGFR between KTRs with and without ADPKD. However, in KTRs with ADPKD, eGFR continued to rise from 1 to 3 years post-transplant, possibly pointing to a higher eGFR in the long-term post-transplant patients compared to those with ESKD secondary to other etiologies (Fig. 2). A lower rate of KTRs with ADPKD were treated with calcium channel blockers compared to those without ADPKD (25.9% vs. 37.7%, *p* = 0.042; Table 2). All other clinical and biochemical characteristics of KTRs with vs. without ADPKD are presented in Table 2.

**Table 2:** Biochemical and clinical characteristics of KTRs stratified by ESKD etiology

Variable	Total cohort (*N* = 513)	ADPKD (*N* = 81)	Other (*N* = 432)	*p*-value
Vital signs and other clinical parameters, 1–12 m-average [mean (SD)]				
SBP (mmHg)	132.3 ± 14.3	129.5 ± 12.6	132.8 ± 14.6	0.05*
DBP (mmHg)	76.8 ± 7.9	79.1 ± 8.5	76.3 ± 7.7	0.005**
Weight (kg)	78.1 ± 15.1	75.9 ± 12.7	78.5 ± 15.5	0.121
BMI (kg/m^2^)	27.1 ± 4.7	25.9 ± 3.7	27.3 ± 4.8	0.012*
Serum creatinine (mg/dL) and eGFR (ml/min; CKD-EPI) post transplant^a^				
Scr 3-m average (*n* = 484) [mean (SD)]	1.3 ± 0.4	1.2 ± 0.4	1.3 ± 0.4	0.038*
eGFR 3-m average (*n* = 484) [mean (SD)]	62.9 ± 18.1	65.2 ± 17.8	62.5 ± 18.1	0.118
Scr 6-m average (*n* = 489) [mean (SD)]	1.3 ± 0.4	1.2 ± 0.4	1.3 ± 0.4	0.156
eGFR 6-m average (*n* = 489) [mean (SD)]	63.2 ± 17.4	64.1 ± 18.2	63.0 ± 17.2	0.308
Scr 1-y average (*n* = 453) [mean (SD)]	1.2 ± 0.3	1.2 ± 0.4	1.2 ± 0.3	0.17
eGFR 1-y average (*n* = 453) [mean (SD)]	65.2 ± 17.8	66.6 ± 19.2	64.9 ± 17.5	0.225
Scr 3-y average (*n* = 273) [mean (SD)]	1.3 ± 0.4	1.2 ± 0.5	1.3 ± 0.4	0.321
eGFR 3-y average (*n* = 273) [median (IQR)]	63.1 (50.3–76.7)	65.3 (55.1–78.1)	63.0 (49.7–76.6)	0.82
Scr 5-y average (*n* = 194) [mean (SD)]	1.4 ± 0.5	1.2 ± 0.4	1.4 ± 0.5	0.041*
eGFR 5-y average (*n* = 194) [median (IQR)]	60.1 (47.5–74.8)	61.8 (55.1–82.9)	58.6 (46.2–74.5)	0.147
Scr 10-y average (*n* = 113) [mean (SD)]	1.3 ± 0.5	1.1 ± 0.4	1.3 ± 0.5	0.056
eGFR 10-y average (*n* = 113) [median (IQR)]	71.7 (48.1–83.8)	77.3 (60.2–89.7)	70.9 (47.1–82.4)	0.165
Scr 3–12 m max, *n* = 508) [mean (SD)]	1.6 ± 0.8	1.4 ± 0.6	1.6 ± 0.8	0.082
eGFR 3–12 m max (*n* = 508) [median (IQR)]	52.0 (40.3–64.3)	56.9 (43.5–66.8)	51.4 (40.2–62.6)	0.092
Scr 3–12 m min (*n* = 508) [mean (SD)]	1.1 ± 0.3	1.0 ± 0.3	1.1 ± 0.3	0.084
eGFR 3–12 m min (*n* = 508) [median (IQR)]	73.4 (62.7–88.0)	74.8 (61.7–90.7)	73.1 (62.8–87.6)	0.649
Scr 3–12- m average (*n* = 508) [mean (SD)]	1.3 ± 0.4	1.2 ± 0.4	1.3 ± 0.4	0.07
eGFR 3–12 m average (*n* = 508) [median (IQR)]	62.6 (50.6–74.5)	65.4 (51.5–78.3)	62.1 (50.5–74.0)	0.267
Urine protein/creatinine ratio 3–12 month average (*n* = 227) [median (IQR)]	0.2 (0.1–0.3)	0.1 (0.1–0.2)	0.2 (0.1–0.3)	0.012*
Medication**s**				
Tacrolimus 3–12 m max (μg/L) [mean (SD)]	12.2 ± 3.7	11.8 ± 3.1	12.3 ± 3.8	0.136
Tacrolimus 3–12 m min (μg/L) [mean (SD)]	5.3 ± 1.7	5.4 ± 1.7	5.3 ± 1.7	0.194
Tacrolimus 3–12 m average (μg/L) [mean (SD)]	8.1 ± 1.4	8.1 ± 1.5	8.1 ± 1.4	0.489
Tacrolimus1-12 m (%)	473 (92.2%)	78 (96.3%)	395 (91.4%)	0.134
Cyclosporine 1–12 m (%)	37 (7.2%)	2 (2.5%)	35 (8.1%)	0.072
MPA 1–12 m(%)	344 (67.1%)	58 (71.6%)	286 (66.2%)	0.343
Prednisone 1–12 m (%)	279 (54.4%)	44 (54.3%)	235 (54.4%)	0.99
mTor inhibitor 1–12 m (%)	12 (2.3%)	4 (4.9%)	8 (1.9%)	0.105
Iron per os 1–12 m (%)	71 (13.8%)	10 (12.3%)	61 (14.1%)	0.671
BB 1–12 m (%)	240 (46.8%)	34 (42.0%)	206 (47.7%)	0.345
CCB 1–12 m (%)	184 (35.9%)	21 (25.9%)	163 (37.7%)	0.042*
RAAS inhibitor 1–12 m (%)	111 (21.6%)	21 (25.9%)	90 (20.8%)	0.307
Azathioprine 1–12 m (%)	2 (0.4%)	0	2 (0.5%)	1
Death (*n* = 513) (%)	75 (14.6%)	4 (4.9%)	71 (16.4%)	0.007**
Time to death (*n* = 71) [mean (SD)]	8.0 ± 4.3	9.8 ± 5.4	7.9 ± 4.2	0.204
Allograft loss (*n* = 513) (%)	30 (5.8%)	4 (4.9%)	26 (6.0%)	0.704
Time to allograft loss (*n* = 30) [mean (SD)]	7.4 ± 4.6	5.0 ± 2.9	7.8 ± 4.8	0.138

^a^ 3-month (m) average, values measured 2.5–3.5 months post-transplant, 6-month average, values measured 5–7 months post-transplant; 1-year average, values measured 10–14 months post-transplant; 3-year average, values measured 33–39 months post-transplant, 5-year average, values measured 54–66 months post-transplant; 10-year average, values measured 114–126 months post-transplant

*BB*, beta blockers; *BMI*, body mass index; *CCB* calcium channel blockers; *CKD-EPI* chronic kidney disease epidemiology collaboration; *DBP* diastolic blood pressure; *eGFR* estimated glomerular filtration rate; *ESKD* end stage kidney disease; *IQR* interquartile range; *MPA* mycophenolic acid; mTOR mammalian target of rapamycin; *RAAS* renin angiotensin aldosterone system; *KTRs* kidney transplant recipients; *Scr* serum creatinine; *SBP* systolic blood pressure; *SD* standard deviation

^***^eGFR was calculated according to the following CKD-EPI formula:

eGFR = 141* min (Scr/k, 1)α * max(Scr/k, 1)− 1.209 * 0.993Age * 1.018 * 1.159 (if black)

(where *Scr* standardized serum creatinine; *k* = 0.7 if female, 0.9 if male; *α* = − 0.329 if female, − 0.411 if male; min = the minimum of Scr/k of 1; max = the maximum of Scr/k or 1)

*p < 0.05; **p < 0.01

**Fig. 3:**
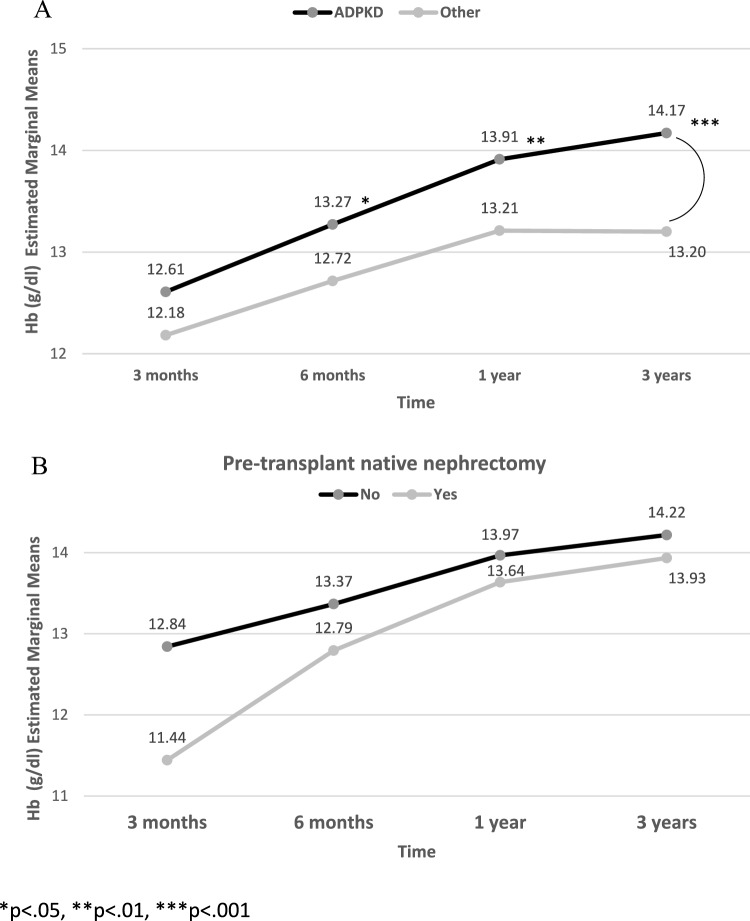
Repeated measurement analysis for Hb levels post-transplant in **A** KTRs with ADPKD versus other etiologies and **B** KTRs with ADPKD who had undergone pre-transplant native nephrectomy versus those who had not. *p < 0.05, **p < 0.01, ***p < 0.001

Hemoglobin levels at different time points post-transplant (averages for 0–3 months, 3 months, 6 months, and 1 year, and 3- to 12-month maximum, minimum and average) were all significantly higher in KTRs with vs. without ADPKD. In a repeated measurements analysis adjusted for multiple comparisons, mean hemoglobin was found to be higher in KTRs with vs. without ADPKD at 6 months, 1 year and 3 years post-transplant (Fig. 3A).

Among the other biochemical parameters related to hemoglobin levels, absolute neutrophil count (3-to 12-month average), ferritin, lactate dehydrogenase, and globulins were lower in KTRs with ADPKD compared to those with ESKD secondary to other etiologies (Table 3).

**Table 3:** Biochemical characteristics, including Hb, Hct and variables related to Hb levels in KTRs stratified by ESKD etiology

Variable	Total cohort (*N* = 513)	ADPKD (*N* = 81)	Other (*N* = 432)	*p*-value
Hemoglobin (g/dL)^a^ and hematocrit (%)				
Hb 0–3 m average	11.1 ± 1.5	11.4 ± 1.5	11.0 ± 1.5	0.019*
Hct 0–3 m average	33.7 ± 4.6	34.6 ± 4.9	33.5 ± 4.5	0.025*
Hb 3-m average	12.3 ± 1.6	12.8 ± 1.9	12.2 ± 1.5	0.005**
Hb 6-m average	12.9 ± 1.6	13.5 ± 1.7	12.8 ± 1.6	< .001**
Hb 1-year average	13.4 ± 1.6	13.9 ± 1.6	13.3 ± 1.6	0.001**
Hb 3–12 m maximum	14.0 ± 1.6	14.6 ± 1.9	13.9 ± 1.5	0.002**
Hb 3–12 m minimum	11.7 ± 1.8	12.2 ± 2.1	11.6 ± 1.8	0.005**
Hb 3–12 m average	12.8 ± 1.6	13.4 ± 1.8	12.7 ± 1.5	< .001**
Hct 3–12 m maximum	42.6 ± 5.0	44.1 ± 5.7	42.2 ± 4.8	0.001**
Hct 3–12 m minimum	35.4 ± 5.6	37.0 ± 6.3	35.1 ± 5.4	0.003**
Hct 3–12 m average	38.9 ± 4.8	40.6 ± 5.4	38.6 ± 4.6	< .001**
Other laboratory results				
MCV 3–12 m average (fL)	88.4 ± 5.7	88.9 ± 4.5	88.4 ± 5.9	0.233
MCH 3–12 m average (pg)	29.2 ± 2.2	29.3 ± 1.7	29.2 ± 2.3	0.354
MCHC 3–12 m average (g/dL)	33.0 ± 0.8	33.0 ± 0.7	33.0 ± 0.9	0.22
WBC 3–12 m average (K/μL)	6.6 ± 2.3	6.5 ± 2.3	6.6 ± 2.3	0.385
Lymphocytes 3–12 m average (K/μL)	1.5 ± 0.6	1.5 ± 0.6	1.5 ± 0.6	0.112
Neutrophils 3–12 m average (K/μL)	4.7 ± 1.7	4.1 ± 1.4	4.8 ± 1.7	< .001**
Platelets 3–12 m average (K/μL)	203.9 ± 61.7	197.3 ± 50.1	205.1 ± 63.6	0.112
Iron blood 1–12 m average (mcg/dL)	62.6 ± 28.6	61.8 ± 21.8	62.8 ± 21.8	0.414
Transferrin 1–12 m average (mg/dL)	194.4 ± 41.8	196.8 ± 36.4	193.9 ± 42.7	0.343
Ferritin 1–12 m average (ng/dL)	319.2 (143.3–660.6)	206.5 (79.1–454.6)	347.9 (167.8–723.3)	0.002**
Vitamin B12 1–12-m average (pmol/L)	381.0 (277.6–547.7)	365.0 (260.7–510.0)	385.2 (279.7–563.5)	0.356
Folic acid 1–12 m average (ng/mL)	10.2 ± 5.2	10.1 ± 4.6	10.2 ± 5.3	0.433
LDH 3–12 m average (n = 501) (U/L)	238.4 ± 53.4	228.5 ± 45.5	240.3 ± 54.6	0.034*
Bilirubin total 3–12 m average (mg/dL)	0.6 ± 0.2	0.6 ± 0.2	0.6 ± 0.2	0.49
TSH 3–12 m average (uIU/mL)	1.5 (1.1–2.5)	1.6 (1.1–2.2)	1.5 (1.0–2.6)	0.9
Albumin 3–12 m average (g/dL)	4.1 ± 0.3	4.1 ± 0.4	4.1 ± 0.3	0.105
Globulin 3–12 m average (g/dL)	2.6 ± 0.4	2.5 ± 0.3	2.6 ± 0.4	< .001**

^a^3-month (m) average, values measured 2.5–3.5 months post-transplant, 6-month average, values measured 5–7 months post-transplant; 1-year average, values measured 10–14 months post-transplant;

*ESKD* end stage kidney disease; *Hb* hemoglobin; *Hct* hematocrit; *LDH* lactate dehydrogenase; *MCV* average mean corpuscular volume; *MCH* average mean corpuscular hemoglobin; *MCHC* mean corpuscular hemoglobin concentration; *KTRs* kidney transplant recipients; *TSH* thyroid stimulating hormone; *WBC* white blood cells

^*^p < 0.05; ^**^p < 0.01

### Linear regression analysis for mean hemoglobin at 3–12 months post-transplant in KTRs

In a multivariable linear regression analysis, mean hemoglobin at 3–12 months post-transplant was higher by 0.76 (standard deviation [SD], 0.17) in ADPKD patients compared to all other KTRs (*p* < 0.001). Mean hemoglobin was found to be higher in males compared to females by 1.06 (SD, 0.14, p < 0.001) and increased by 0.03 (SD, 0.00) for every increase of 1 ml/min in average eGFR at 3–12 months post-transplant (*p* < 0.001; Table 4).

**Table 4:** Multivariate linear regression analysis for mean Hb 3–12 months post-transplant in KTRs (*N* = 505)

Effect	Mean (SD)	*p*-value
ADPKD vs. all other ESKD etiologies	0.76 (0.17)	< 0.001**
Age on transplant date, per 1 year increase	-0.01 (0.00)	0.09
Sex, male vs. female	1.06 (0.14)	< 0.001**
HTN, yes vs. no	0.22 (0.14)	0.13
Pretransplant diabetes mellitus, yes vs. no	-0.26 (0.19)	0.17
IHD, yes vs. no	0.37 (0.22)	0.09
eGFR 3–12 m average, per 1 ml/min increase	0.03 (0.00)	< 0.001**
Neutrophils 3–12 m average, per 1 K/μL increase	0.06 (0.04)	0.1

^**^p < 0.01

### Logistic regression analysis for maximal hemoglobin at 3–12 months post-transplant > 15 mg/dL in KTRs

In a multivariable logistic regression analysis, the odds for maximum hemoglobin to be > 15 mg/dL at 3–12 months post-transplant were more than twice as high in ADPKD KTRs versus all other KTRs [odds ratio (OR) 2.31, 95% confidence interval (CI) 1.3–4.13, *p* < 0.001]. Age and male sex were also found to be independent predictors for maximum hemoglobin > 15 mg/dL. A medical history of hypertension increased the odds for maximum hemoglobin > 15 mg/dL by 87% [OR 1.87, 95% CI 1.14–3.08, *p* = 0.01]. A higher level of absolute neutrophils was also found to be an independent predictor for maximum hemoglobin > 15 mg/dL, with the *p* value approaching significance (Table 5).

**Table 5:** Multivariate logistic regression for max Hb > 15 mg/dL 3–12 months post-transplant in KTRs (*N* = 505)

Effect	Odds ratio (95% CI)	*p*-value
ADPKD vs. other	2.31 (1.30–4.13)	< 0.001**
Age at transplant, per 1 year increase	0.98 (0.96–1.00)	0.03*
Male vs. female	3.99 (2.20–7.22)	< 0.001**
HTN, yes vs no	1.87 (1.14–3.08)	0.01*
Pretransplant diabetes mellitus, yes vs. no	0.79 (0.41–1.54)	0.5
IHD, yes vs. no	1.17 (0.57–2.40)	0.67
eGFR (CKD-EPI) 3–12 months, per 1 ml/min increase	1.01 (1.00–1.02)	0.11
Neutrophils 3–12 m average, per 1 K/μL increase	1.13 (1.00–1.29)	**0.05**

*CKD-EPI* chronic kidney disease epidemiology collaboration; *eGFR* estimated glomerular filtration rate; *ESKD* end stage kidney disease; *Hb* hemoglobin; *HTN* hypertension; *IHD* ischemic heart disease; *ADPKD* autosomal dominant polycystic kidney disease; *KTRs* kidney transplant recipients

^*^p < 0.05; ^**^p < 0.01

### Subgroup analysis for hemoglobin in KTRs with ADPKD divided into those who had undergone pre-transplant native nephrectomy and those who had not

The KTRs in the ADPKD group (*n* = 81) were divided into those who had undergone pre-transplant native nephrectomy [*n* = 20, made up of unilateral (*n* = 15), bilateral (*n* = 5)] and those who had not. There were no statistically significant differences in hemoglobin levels at different time points post-transplant, but there was a tendency for lower hemoglobin levels in those who had undergone unilateral or bilateral pre-transplant native nephrectomy compared to those who had not (Table 6). A repeated measurement analysis adjusted for multiple comparisons also showed this tendency (Fig. 3B).

**Table 6:** Univariate analysis for Hb (g/dL) in KTRs with ADPKD divided into KTRs who underwent pre-transplant native nephrectomy and those who did not [mean (SD)]

	All ADPKD cohort (*n* = 81)	Yes (*n* = 20)	No (*n* = 61)	*p*- value
Hb 3-month average^a^	12.8 ± 1.9	12.4 ± 1.7	13.0 ± 2.0	0.124
Hb 6-month average^a^	12.8 ± 1.9	13.2 ± 1.4	13.5 ± 1.8	0.197
Hb 3–12 month max	14.6 ± 1.9	14.5 ± 2.0	14.6 ± 1.9	0.422
Hb 3–12 month min	12.2 ± 2.1	11.9 ± 1.8	12.3 ± 2.1	0.224
Hb 3–12 month average	13.4 ± 1.8	13.1 ± 1.4	13.5 ± 1.9	0.229
Hb 3-year average^a^	14.0 ± 1.7	13.6 ± 1.6	14.1 ± 1.8	0.197

^a^ 3-month average, values measured 2.5–3.5 months post-transplant, 6-month average, values measured 5–7 months post-transplant; 3-year average, values measured 33–39 months post-transplant

*Hb* hemoglobin; *ADPKD* autosomal dominant polycystic kidney disease; *KTRs* kidney transplant recipients; *SD* standard deviation

### Linear Regression Analysis for eGFR at 1 Year Post-Transplant in KTRs

In a multivariable linear regression analysis for eGFR at 1 year post-transplant, mean eGFR was higher in men by 3.778 ml/min (SD 1.704, *p* = 0.027). For every increase of 1 g/dL in average hemoglobin at 3–12 months post-transplant, mean eGFR increased by 1.456 ml/min (SD 0.467, *p* = 0.002). Estimated GFR at 3 months post-transplant was found to be an independent predictor for eGFR at 1 year post-transplant, as mean eGFR increased by 0.786 ml/min (SD 0.04) for every increase of 1 ml/min in eGFR at 3 months post-transplant (*p* < 0.001). ESKD secondary to ADPKD as well as pre-transplant diabetes, ischemic heart disease, BMI, systolic blood pressure and neutrophil count were not found to be independently associated with eGFR at 1 year post-transplant (Table 7).

**Table 7:** Multivariable linear regression analysis for eGFR at 1 year post-transplant (n = 260)

Effect	Mean (SD)	*p*-value
Age, per 1 year increase	– 0.062 (0.56)	0.265
Sex, male vs. female	3.778 (1.704)	0.027*
ADPKD vs. all other ESKD etiologies	0.651 (2.006)	0.746
Pretransplant diabetes mellitus, yes vs. no	2.264 (1.735)	0.193
IHD, yes vs. no	0.935 (1.971)	0.636
BMI 1–12 month average, per 1 kg/m^2^ increase	0.006 (0.154)	0.968
SBP 1–12 month average, per 1 mmHg increase	– 0.027 (0.055)	0.621
Hb 3–12 month average, per 1 g/dL increase	1.456 (0.467)	0.002**
Neutrophils 3–12 month average, per 1 K/μL increase	– 0.286 (0.428)	0.505
eGFR 3-month average, per 1 ml/min increase	0.786 (0.040)	< .001**

*BMI* body mass index; *eGFR* estimated glomerular filtration rate; *ESKD* end stage kidney disease; Hb, hemoglobin; *IHD* ischemic heart disease; *ADPKD* autosomal dominant polycystic kidney disease; *SBP* systolic blood pressure; *SD* standard deviation

^*^p < 0.05; ^**^p < 0.01

### Long-term patient and allograft survival in KTRs with ADPKD vs. those with ESKD due to other conditions

**Fig. 4:**
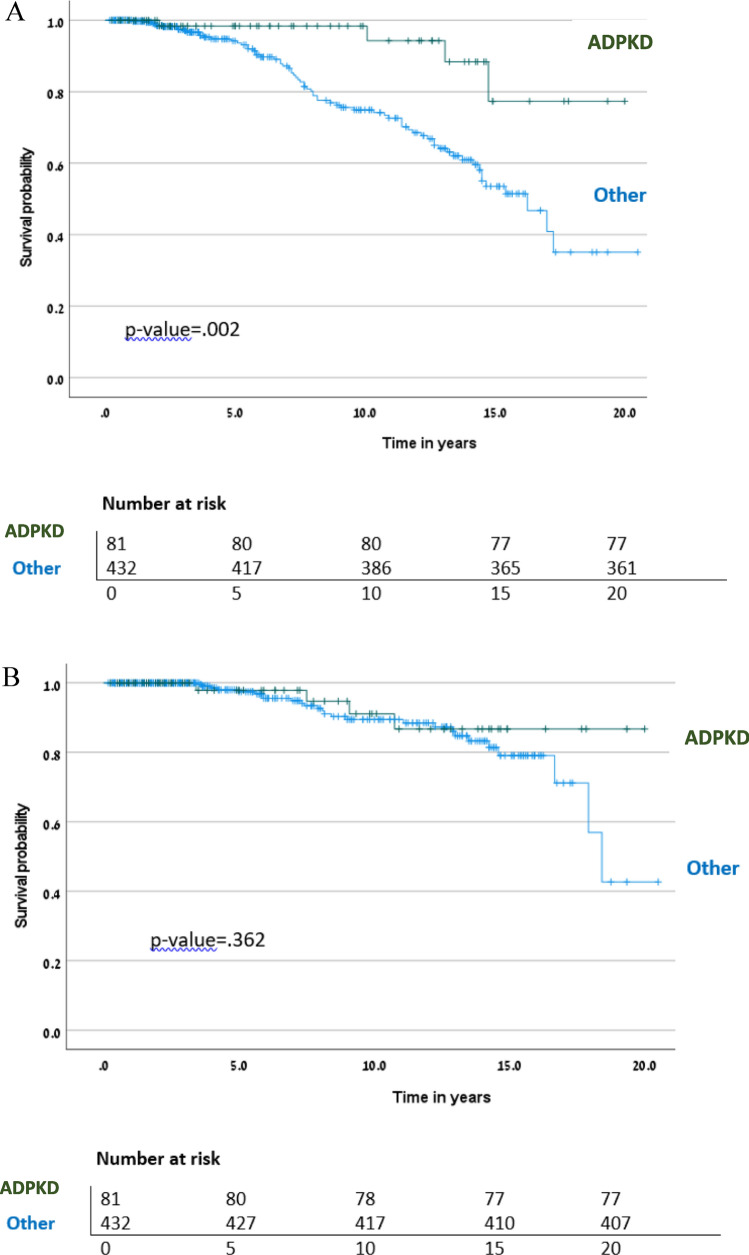
Kaplan Meier survival analysis for **A**: patient survival in KTRs with ADPKD versus others, and **B** death-censored allograft survival in KTRs with ADPKD versus others

Patient survival increased over a 20-year span in KTRs with ADPKD, in comparison to those with ESKD stemming from other causes (Fig. 4A,* p* = 0.002). However, there did not appear to be a significant difference in death-censored allograft survival between the two groups, possibly due to the limited instances of allograft loss (Fig. 4B, *p* = 0.362). In a multivariable Cox regression hazard model for patient survival, mortality risk was reduced by 68% in KTRs with ADPKD compared to those with other ESKD causes [HR 0.32, 95% CI, 0.12–0.88, *p* = 0.03]. Ischemic heart disease and neutrophil count were also found to be independent predictors for morality (Table 8).

**Table 8:** Cox regression hazard model for mortality (*N* = 480)

Effect	HR (95% CI)	*p-*value
Age, per 1 year increase	1.06 (1.04–1.09)	< 0.001**
Sex, male vs. female	1.52 (0.81–2.88)	0.20
ADPKD vs. all other ESKD etiologies	0.32 (0.12–0.88)	0.03*
Pretransplant diabetes mellitus, yes vs. no	0.51 (0.14–1.87)	0.31
IHD, yes vs. no	4.55 (1.38–15.0)	0.01*
Hb 3–12 month average, per 1 g/dL increase	0.88 (0.73–1.06)	0.17
Neutrophils 3–12 month average, per 1 K/μL increase	1.19 (1.02–1.40)	0.03*
eGFR 3-month average, per 1 ml/min increase	1.01 (0.99–1.03)	0.38

*CI* confidence interval; *eGFR* estimated glomerular filtration rate; *ESKD* end stage kidney disease; Hb, hemoglobin; *HR* hazard ratio; *IHD* ischemic heart disease; *ADPKD* autosomal dominant polycystic kidney disease

^*^p < 0.05; ^**^p < 0.01

## Discussion

**Fig. 5:**
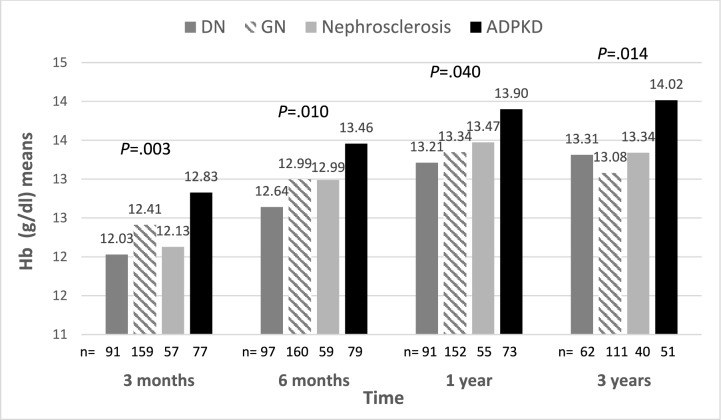
Mean Hb levels post-transplant in different ESKD groups. *DN* diabetic nephropathy; *GN* glomerulonephritis

After conducting a comprehensive comparison of a range of clinical and biochemical traits between KTRs with ADPKD and those with ESKD originating from other factors, this study identified a noteworthy difference in hemoglobin levels between the two groups of patients, namely, significant differences in hemoglobin levels between the two groups were observed as the duration following transplantation continued, up to as long as three years post-transplant (Fig. 5). Furthermore, ADPKD emerged as an autonomous predictor for elevated hemoglobin levels, as confirmed by multivariate analyses. These findings align with previous studies indicating a positive association between hemoglobin levels and the presence of polycystic kidneys [10] and a higher incidence of post-transplantation erythrocytosis in KTRs with ADPKD [7, 8].

To elucidate the mechanisms underpinning the elevated erythrocytosis in KTRs with ADPKD compared to other KTRs, we conducted a subgroup analysis. Within the KTRs with ADPKD group, individuals were categorized based on whether they had undergone pre-transplant native nephrectomy or not. Owing to a limited number of participants who had undergone native nephrectomy – whether unilateral or bilateral – we were unable to show a statistically significant difference in hemoglobin levels between the two subgroups. Nonetheless, a clear differentiation became apparent at various time points post-transplant when the results were assessed through both univariate and multiple comparison analyses. This finding underscores the potential role of the native kidneys in ongoing erythropoietin production, which seems to persist for years following transplantation in KTRs with ADPKD. The existing literature indicates that approximately 20–30% of patients with ADPKD undergo native nephrectomy [11–13]. In a previous sole study that investigated 33 patients with ADPKD who had undergone bilateral nephrectomy, a marked reduction in hemoglobin levels was observed post-nephrectomy. This decrease was evident both in patients who had received a kidney transplant and the group undergoing dialysis [14]. Importantly, our study uniquely shows a difference in hemoglobin levels within the specific subset of KTRs with ADPKD, stratified based on the presence or absence of pre-transplant nephrectomy.

Analysis of cyst fluid from 18 ADPKD patients showed a positive correlation between the erythropoietin content of the cysts and the hormone levels in the serum, indicating that the erythropoietin level in the cysts reflects the kidneys' erythropoietin production capacity. Interstitial cells adjacent to proximal tubular cysts in ADPKD patients produce erythropoietin independently of the oxygen pressure or the pH of the cyst fluid, two physiological parameters that affect erythropoietin production in the normal kidney. This activity contributes to the higher levels of hemoglobin observed in patients with end-stage ADPKD [6]. Hypoxia-inducible factor-2α, a hypoxia-inducible transcription factor, is expressed in peri-cystic stromal and endothelial cells in the cystic kidney tissue. This factor is activated by regional hypoxia and is linked to activation of erythropoietin in ADPKD patients [15]. Our findings suggest that the mechanisms governing erythropoietin production in native polycystic kidneys continue to function following renal transplantation.

Prior studies have demonstrated a strong association between hemoglobin and renal allograft function [16, 17]. Moreover, long-term patient and graft survival was enhanced in KTRs with ADPKD compared to those with other ESKD etiologies [18]. In a comprehensive multivariable analysis encompassing comparisons between KTRs with ADPKD and those without, along with other variables exhibiting significant differences between the two groups, our findings revealed that the sole independent predictors for eGFR at 1 year post-transplant were hemoglobin levels, male sex, and eGFR at 3 months post-transplant. This observation sheds light on the underlying mechanism accountable for the superior outcomes witnessed in KTRs with ADPKD in relation to improved renal allograft function, patient longevity, and allograft survival.

Although we did not find a statistically significant difference in renal allograft function across various post-transplant time points between KTRs with and without ADPKD, we did show a trend towards an improved eGFR in the KTRs with ADPKD over time. Moreover, in comparison to other KTRs, individuals with ADPKD exhibited higher long-term patient survival rates. These findings underscore the pivotal role played by elevated hemoglobin levels in KTRs with ADPKD, which probably contributes to the better outcomes observed post-transplant.

Of note, our investigation revealed lowered levels of neutrophils, ferritin, lactate dehydrogenase, and globulins in KTRs with ADPKD in contrast to those with ESKD stemming from other causes. Additionally, the higher neutrophil count increased the hazard for mortality. These observations could potentially signify a lowered state of inflammation in KTRs with ADPKD when compared to their counterparts. This decrease in inflammation-associated conditions [19, 20] might offer an explanation for the observed enhancement in overall physical well-being, coupled with reduced morbidity and mortality in these patients. Interestingly, our analysis revealed an association between a medical history of hypertension and elevated hemoglobin levels. This observation could potentially be attributed to the involvement of hypoxia-inducible factors in biological mechanisms involved in blood pressure regulation and erythropoietin production [21].

Potential limitations should be mentioned when interpreting the study results, including the retrospective design, potential confounding variables, and single-center focus. Variability in the methods used to measure certain variables (e.g., hemoglobin levels, inflammatory markers) across different time points and between different patients could introduce measurement bias and affect the accuracy of the results. Importantly, while associations between variables were observed, the study was not able to establish a cause-and-effect relationship due to its observational nature. An additional limitation was the small sample sizes in the subgroup analyses, such as those involving patients with ADPKD who had undergone nephrectomy and those who had not, which could affect the statistical power of these analyses. However, our study focused on a specific subset of KTRs with ADPKD, allowing for an in-depth analysis of their characteristics compared to other recipients, while using a wide range of clinical, biochemical, and demographic variables. The other strengths of the study included an examination of outcomes over different time points following transplantation, which enabled both the observation of trends and changes over an extended post-transplant period and an investigation of a unique subgroup of KTRs with ADPKD who had undergone pre-transplant nephrectomy.

In summary, hemoglobin levels were elevated in KTRs with ADPKD relative to their ESKD peers. This phenomenon could be attributed to the enduring augmentation of erythropoietin production by the native kidneys years after transplantation. Notably, these heightened hemoglobin levels play a pivotal role in driving the observed enhanced outcomes, underlining their significance in shaping the overall success of transplantation in this particular patient subset. The improved patient survival and potential renal allograft benefits observed in KTRs with ADPKD highlight the importance of recognizing ADPKD as a distinct subset in the context of transplantation outcomes. Furthermore, this study opens avenues for future research to unravel the underlying mechanisms that differentiate KTRs with ADPKD from their counterparts.

## Data Availability

The data underlying this article will be shared on reasonable request to the corresponding author.

## Abbreviations

BB: Beta blockers
BMI: Body mass index
CCB: Calcium channel blockers
CHF: Congestive heart failure
CI: Confidence interval
CKD: Chronic kidney disease
CKD-EPI: Chronic kidney disease epidemiology collaboration
CNI: Calcineurin inhibitor
DBP: Diastolic blood pressure
DN: Diabetic nephropathy
eGFR: Estimated glomerular filtration rate
EPO: Erythropoietin
ESKD: End-stage kidney disease
GN: Glomerulonephritis
Hb: Hemoglobin
Hct: Hematocrit
HIF: Hypoxia-inducible factor
HTN: Hypertension
HR: Hazard ratio
IHD: Ischemic heart disease
IQR: Interquartile range
KTR: Kidney transplant recipients
LDH: Lactate dehydrogenase
MCV: Mean corpuscular volume
MCH: Mean corpuscular hemoglobin
MCHC: Mean corpuscular hemoglobin concentration
MPA: Mycophenolic acid
mTOR: Mammalian target of rapamycin inhibitors
OR: Odds ratio
ADPKD: Autosomal dominant polycystic kidney disease
PTE: Post-transplantation erythrocytosis
RAAS: Renin angiotensin aldosterone system
SBP: Systolic blood pressure
Scr: Serum creatinine
SD: Standard deviation
TSH: Thyroid stimulating hormone
WBC: White blood cells

## References

[1] Wilson PD (2004) Polycystic kidney disease. N Engl J Med 350(2):151–164 14711914 10.1056/NEJMra022161

[2] Mochizuki T, Tsuchiya K, Nitta K (2013) Autosomal dominant polycystic kidney disease: recent advances in pathogenesis and potential therapies. Clin Exp Nephrol 17(3):317–326 23192769 10.1007/s10157-012-0741-0

[3] Chandra M, Miller ME, Garcia JF, Mossey RT, McVicar M (1985) Serum immunoreactive erythropoietin levels in patients with polycystic kidney disease as compared with other hemodialysis patients. Nephron 39(1):26–29 3969187 10.1159/000183332

[4] de Almeida EA, Alho I, Marques F, Thiran C, Bicho MP, Prata M (2008) Haemoglobin and erythropoietin levels in polycystic kidney disease. Nephrol Dial Transpl 23(1):412–413 10.1093/ndt/gfm717 17951311

[5] Verdalles U, Abad S, Vega A, Ruiz Caro C, Ampuero J, Jofre R et al (2011) Factors related to the absence of anemia in hemodialysis patients. Blood Purif 32(1):69–74 21346339 10.1159/000323095

[6] Eckardt KU, Mollmann M, Neumann R, Brunkhorst R, Burger HU, Lonnemann G et al (1989) Erythropoietin in polycystic kidneys. J Clin Investig 84(4):1160–1166 2794053 10.1172/JCI114280 PMC329773

[7] Hofstetter L, Rozen-Zvi B, Schechter A, Raanani P, Itzhaki O, Rahamimov R et al (2021) Post-transplantation erythrocytosis in kidney transplant recipients-A retrospective cohort study. Eur J Haematol 107(6):595–601 34370889 10.1111/ejh.13696

[8] Mekraksakit P, Boonpheng B, Leelaviwat N, Duangkham S, Deb A, Kewcharoen J et al (2021) Risk factors and outcomes of post-transplant erythrocytosis among adult kidney transplant recipients: a systematic review and meta-analysis. Transpl Int 34(11):2071–2086 34412165 10.1111/tri.14016

[9] Hod T, Ben-David A, Olmer L, Scott N, Ghinea R, Mor E et al (2022) BNT162b2 third booster dose significantly increases the humoral response assessed by both RBD IgG and neutralizing antibodies in renal transplant recipients. Transpl Int 35:10239 35387393 10.3389/ti.2022.10239 PMC8977405

[10] Lorenz M, Kletzmayr J, Perschl A, Furrer A, Horl WH, Sunder-Plassmann G (2002) Anemia and iron deficiencies among long-term renal transplant recipients. J Am Soc Nephrol 13(3):794–797 11856787 10.1681/ASN.V133794

[11] Patel P, Horsfield C, Compton F, Taylor J, Koffman G, Olsburgh J (2011) Native nephrectomy in transplant patients with autosomal dominant polycystic kidney disease. Ann R Coll Surg Engl 93(5):391–395 21943464 10.1308/003588411X582690 PMC3365458

[12] Kanaan N, Devuyst O, Pirson Y (2014) Renal transplantation in autosomal dominant polycystic kidney disease. Nat Rev Nephrol 10(8):455–465 24935705 10.1038/nrneph.2014.104

[13] Darius T, Bertoni S, De Meyer M, Buemi A, Devresse A, Kanaan N et al (2022) Simultaneous nephrectomy during kidney transplantation for polycystic kidney disease does not detrimentally impact comorbidity and graft survival. World J Transplant 12(5):100–111 35663541 10.5500/wjt.v12.i5.100 PMC9136716

[14] Bellini MI, Charalmpidis S, Brookes P, Hill P, Dor F, Papalois V (2019) Bilateral nephrectomy for adult polycystic kidney disease does not affect the graft function of transplant patients and does not result in sensitisation. Biomed Res Int 2019:7423158 31309115 10.1155/2019/7423158 PMC6594324

[15] Buchholz B, Eckardt KU (2020) Role of oxygen and the HIF-pathway in polycystic kidney disease. Cell Signal 69:109524 31904413 10.1016/j.cellsig.2020.109524

[16] Vanrenterghem Y, Ponticelli C, Morales JM, Abramowicz D, Baboolal K, Eklund B et al (2003) Prevalence and management of anemia in renal transplant recipients: a European survey. Am J Transplant Off J Am Soc Transplant Am Soc Transplant Surg 3(7):835–845 10.1034/j.1600-6143.2003.00133.x 12814475

[17] Tsujita M, Kosugi T, Goto N, Futamura K, Nishihira M, Okada M et al (2019) The effect of maintaining high hemoglobin levels on long-term kidney function in kidney transplant recipients: a randomized controlled trial. Nephrol Dial, Transpl 34(8):1409–1416 10.1093/ndt/gfy365 PMC6680099 30561729

[18] Bhutani G, Astor BC, Mandelbrot DA, Mankowski-Gettle L, Ziemlewicz T, Wells SA et al (2021) Long-term outcomes and prognostic factors in kidney transplant recipients with polycystic kidney disease. Kidney360 2(2):312–324 35373032 10.34067/KID.0001182019 PMC8740986

[19] Couzin-Frankel J (2010) Immune therapy steps up the attack. Science 330(6003):440–443 20966228 10.1126/science.330.6003.440

[20] Furman D, Campisi J, Verdin E, Carrera-Bastos P, Targ S, Franceschi C et al (2019) Chronic inflammation in the etiology of disease across the life span. Nat Med 25(12):1822–1832 31806905 10.1038/s41591-019-0675-0 PMC7147972

[21] Afsar B, Afsar RE (2023) Hypoxia-inducible factors and essential hypertension: narrative review of experimental and clinical data. Pharmacol Rep PR 75(4):861–875 37210694 10.1007/s43440-023-00497-x

